# An epidemic of chikungunya in northwestern Bangladesh in 2011

**DOI:** 10.1371/journal.pone.0212218

**Published:** 2019-03-11

**Authors:** Farhana Haque, Mahmudur Rahman, Nuzhat Nasreen Banu, Ahmad Raihan Sharif, Shamim Jubayer, AKM Shamsuzzaman, ASM Alamgir, Jesse H. Erasmus, Hilda Guzman, Naomi Forrester, Stephen P. Luby, Emily S. Gurley

**Affiliations:** 1 Infectious Diseases Division (IDD), icddr,b, Dhaka, Bangladesh; 2 Institute of Epidemiology, Disease Control and Research (IEDCR), Dhaka, Bangladesh; 3 Institute for Translational Sciences, University of Texas Medical Branch, Galveston, Texas, United States of America; 4 Institute for Human Infections and Immunity, University of Texas Medical Branch, Galveston, Texas, United States of America; 5 Department of Pathology, University of Texas Medical Branch, Galveston, Texas, United States of America; 6 Global Disease Detection Branch, Division of Global Health Protection, Center for Global Health, Centers for Disease Control and Prevention (CDC), Atlanta, Georgia, United States of America; Makerere University School of Public Health, UGANDA

## Abstract

**Background:**

In November 2011, a government hospital physician in Shibganj sub-district of Bangladesh reported a cluster of patients with fever and joint pain or rash. A multi-disciplinary team investigated to characterize the outbreak; confirm the cause; and recommend control and prevention measures.

**Methods:**

Shibganj's residents with new onset of fever and joint pain or rash between 1 September and 15 December 2011 were defined as chikungunya fever (CHIKF) suspect cases. To estimate the attack rate, we identified 16 outpatient clinics in 16 selected wards across 16 unions in Shibganj and searched for suspect cases in the 80 households nearest to each outpatient clinic. One suspect case from the first 30 households in each ward was invited to visit the nearest outpatient clinic for clinical assessment and to provide a blood sample for laboratory testing and analyses.

**Results:**

We identified 1,769 CHIKF suspect cases from among 5,902 residents surveyed (30%). Their median age was 28 (IQR:15−42) years. The average attack rate in the sub-district was 30% (95% CI: 27%−33%). The lowest attack rate was found in children <5 years (15%). Anti-CHIKV IgM antibodies were detected by ELISA in 78% (264) of the 338 case samples tested. In addition to fever, predominant symptoms of serologically-confirmed cases included joint pain (97%), weakness (54%), myalgia (47%), rash (42%), itching (37%) and malaise (31%). Among the sero-positive patients, 79% (209/264) sought healthcare from outpatient clinics. CHIKV was isolated from two cases and phylogenetic analyses of full genome sequences placed these viruses within the Indian Ocean Lineage (IOL). Molecular analysis identified mutations in E2 and E1 glycoproteins and contained the E1 A226V point mutation.

**Conclusion:**

The consistently high attack rate by age groups suggested recent introduction of chikungunya in this community. Mosquito control efforts should be enhanced to reduce the risk of continued transmission and to improve global health security.

## Introduction

Epidemics of chikungunya fever (CHIKF) caused by chikungunya virus (CHIKV), a mosquito-transmitted alpha virus of the *Togaviridae* family, have been reported from several countries in Africa, Asia and the Western Pacific since 1952.[1–3] Large outbreaks in the Indian Ocean region and the recent international progression of CHIKV in the Europe and from the Caribbean to the Americas have heightened the global health security concern for chikungunya.[4–12] *Aedes aegypti* and *Aedes albopictus* mosquitoes are the two primary vectors transmitting CHIKV to humans.[3] Classical CHIKF is characterized by a triad of clinical features including fever, polyarthralgia or pain in multiple joints and a rash that usually resolves within 7 days.[5, 13–16] However, joint pain involving the lower limbs can sometimes cause severe disability and rarely persist for weeks to months, or for years.[5, 13, 17–19] Furthermore, atypical rheumatological, neurological, cardiac, ocular and renal manifestations as well as chronic comorbidities and deaths have been reported in the past.[20–30]

During December 2008, CHIKV infection was confirmed for the first time in Bangladesh.[31] The outbreak reportedly affected 39 people who were from the same family and/or neighbourhood in two northwestern districts.[31] Another outbreak with very limited geographic transmission was reported in Shathia sub-district of Pabna district in 2009.[32] Outbreaks were not identified in 2010, but an outbreak was confirmed in four villages of Dohar sub-district within Dhaka district in late October 2011 that had reportedly affected 7% (275/3840) of the inhabitants.[33] Since then several outbreaks have been reported in the country between 2012 and 2017 in Tangail, Dhaka and Sitakundu along with the epidemic transmission of the pathogen in the Dhaka City Corporation Area from May 2017.[32, 34]

On 5 November 2011, the primary healthcare physician managing the government hospital in Shibganj sub-district of Chapainababganj district in northwestern Bangladesh reported a cluster of 19 patients with fever, severe joint pain and/or rash to the Institute of Epidemiology, Disease Control and Research (IEDCR) of the Ministry of Health and Family Welfare. In response, a collaborative, multi-disciplinary investigation team from IEDCR and icddr,b conducted an investigation to (i) characterize the outbreak in terms of person, place and time; (ii) confirm the cause; and (iii) identify prevention and control approaches for future outbreaks.

## Materials and methods

### Epidemiological investigation

#### Preliminary investigation

The team visited the hospital in the affected sub-district and conducted unstructured interviews with local healthcare providers, including physicians and field workers, to collect preliminary information about the clinical features, laboratory findings, and geographical distribution of affected patients. Based on these interviews, the experience of a similar outbreak in 2008,[31] and given that all available antibody test results for dengue virus were negative, the team suspected this event as an outbreak of CHIKF. The team defined a suspect case of CHIKF as any resident of Shibganj sub-district with new onset of fever and joint pain or rash between 1 September and until the date of interview. Using the case definition, the team identified and enlisted suspect cases from among the patients who were seeking outpatient care from the hospital during 15−17 November 2011. Team physicians used a standardized, pretested questionnaire developed for investigating CHIKF outbreaks in the past to collect clinical histories and conducted physical examination. Trained phlebotomists collected 5ml blood from the suspect cases who agreed to provide samples. To assess the potential burden of CHIKF in the community, data collectors were trained to conduct a house-to-house survey in the 50 households nearby the hospital for identifying the suspect cases. The collected sera were tested using a rapid test (SD Bioline Chikungunya IgM, Standard Diagnostics, South Korea) in the Virology Laboratory of IEDCR following the manufacturer's instructions. The manufacturer reported relative sensitivity of the qualitative, immunochromatographic, rapid test was 97% and specificity was 99% when compared to the commercial CHIKV IgM capture ELISA.[35]

#### Syndromic survey to describe the magnitude of the illness outbreak

**Fig 1:**
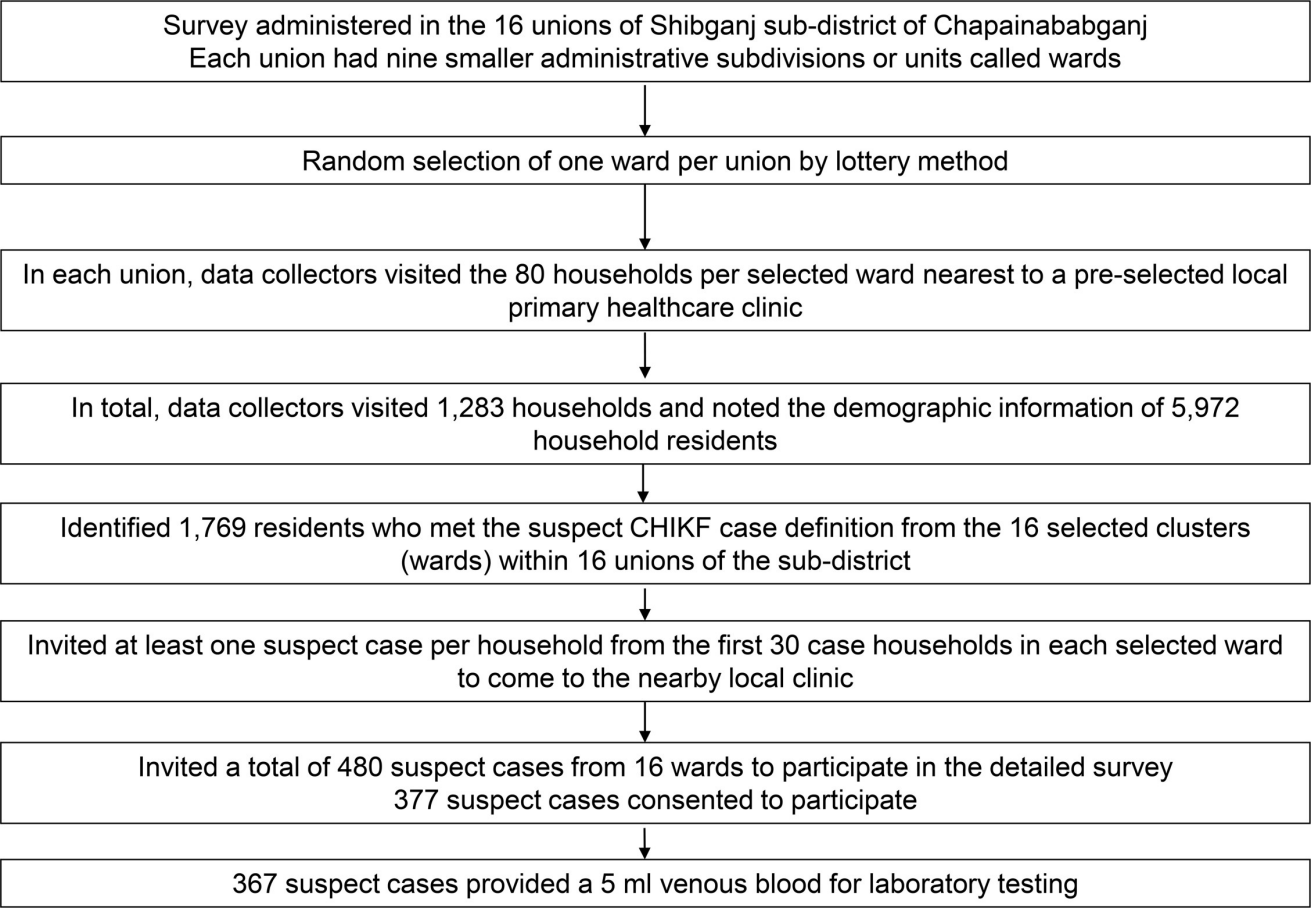
Participant enrollment process for the chikungunya fever (CHIKF) syndromic survey and clinical case survey in Shibganj sub-district of Chapainawabganj district in Bangladesh, 1 September −15 December, 2011.

Given the lack of information regarding the prevalence of chikungunya fever from Bangladesh during the investigation period in 2011 and because the preliminary survey findings suggested a widespread distribution of suspect cases, the investigation team conducted a house-to-house syndromic survey in all of the 16 unions of Shibganj to calculate the attack rate. Using the attack rate of 34% as observed during the preliminary survey and assuming a type 1 error of 0.05, a precision of 5%, an average of 5 persons per household, a design effect of 3.0 to take into account the cluster sampling design and a non-response rate of 20% at the household level, we calculated a required sample size of at least 1,280 households from the 16 unions of Shibganj for the syndromic survey.[36] In each union, we randomly selected one out of the nine administrative wards for the survey. In each selected ward within a union, we identified a local primary healthcare clinic. We defined the 80 households nearest to each selected clinic as a cluster. Five trained data collectors visited the 80 households nearby the selected clinic within the cluster in each selected ward within a union. In the three unions where wards with primary healthcare clinics could not be identified, data collectors started household enrollment from a school or a mosque instead. In each household visited, data collectors collected the age and sex of all households members, and noted if any household resident met the definition of a suspect CHIKF case. Given that the majority of the suspect cases could only recall the month of symptom onset and failed to reliably report the exact date of illness onset, data collectors did not ask about the date of illness onset during the community survey (Fig 1).

#### Clinical survey

To obtain clinical histories and collect biological specimens, data collectors invited one suspect case from each of the first 30 households visited in each ward to come to the nearest primary healthcare clinic to complete a clinical assessment and to provide a blood sample for testing. If a household had more than one eligible respondent, the data collectors requested the eldest household member present during the survey to select one resident for participation. Physicians on the team later interviewed and physically examined suspect cases who came to the local clinics.

### Laboratory investigations

#### Serologic identification

To test for IgM antibodies to CHIKV, trained phlebotomists collected 5 ml venous blood from each suspect case who had presented to the clinic and consented to participate. Phlebotomists stored all blood samples immediately after collection at 4−8°C. The samples were centrifuged on the same day and the sera were stored in the local hospital refrigerator and later transported in cool boxes to the Virology Laboratory of IEDCR. At IEDCR, each serum sample was divided into three aliquots before storing at -70°C until testing. At the beginning of the investigation, we only had 300 rapid test kits available, so we tested 300 randomly selected samples out of the 367 sera. We tested 338 randomly selected aliquots out of the 367 samples for anti-CHIKV immunoglobulin M (IgM) antibodies using an enzyme-linked immunosorbent assay (ELISA) test kit that was also available with our group during the investigation (Bioline Chikungunya IgM manufactured by Standard Diagnostics Inc., Yongin-si,South Korea) in the Virology Laboratory of IEDCR following the manufacturer’s guidelines.[37]

#### Virus isolation and sequencing

Acute sera that had been collected from suspect cases within two days of illness onset and tested negative for IgM antibodies to CHIKV were later tested for viral RNA using quantitative reverse transcription polymerase chain reaction (RT-qPCR).[38] The sera were inoculated onto Vero cell cultures and monitored for cytopathic effects. Virus-positive cell-supernatants were passed on Vero cells in a T-150 cell culture flask, cell supernatant was clarified at 48 hours post-infection, virus was concentrated by polyethylene glycol precipitation, and RNA was trizol-extracted as previously described.[39] Viral RNA was then sequenced by illumina next-generation sequencing and full-length genomes were assembled using the template assembly implemented in the NGen program from Lasergene (DNAStar, Madison, WI). All changes from the template were manually verified and the assembly were checked for sequencing errors.

#### Phylogenetic analysis

Phylogenetic analysis was performed as previously described.[40] Chikungunya full-genome nucleotide sequences were downloaded from the GenBank and aligned using MUSCLE.[41] Individual genomes were then added and aligned manually to preserve the previous alignment. Maximum-likelihood analysis was performed and the modelTest in Phylogenetic Analysis Using Parsimony (PAUP) was used to determine the best-fit nucleotide substitution model, GTR+I+G.[42] The robustness of the maximum-likelihood phylogeny was evaluated by performing the bootstrap resampling of 100 generations.

### Entomological investigation

The entomological investigation was conducted during December 15−31, 2011 and focused on identifying vector species. Given that *Aedes aegypti* bred mainly in the water collected in artificial containers in the environment of human settlements in Asia,[43] while *Aedes albopictus* bred predominantly in natural containers including coconut shells, leaf axils and tree holes, team entomologists looked for mosquito larvae in both man-made and natural water containers.[44] To collect mosquito larvae, entomologists visited 10 households starting from the suspect case household nearest to the local clinic and until reaching nine other case households within a 400-metre radius from the first surveyed household in each of the 16 wards where the syndromic survey had been administered. After visiting each household, entomological team members explained the larvae collection process to household residents and sought verbal informed consent from the household head or the eldest resident present to search the premises and adjacent area and collect mosquito larvae. The World Health Organization’s vector surveillance guidelines were followed to transport larvae in glass vials to the IEDCR's Entomology Laboratory and to determine the mosquito species after hatching.[45]

### Data analysis

We divided the total number of suspect cases by the total number of household residents surveyed in each cluster ward within each union composed of the 80 households nearest to an outpatient clinic in the ward to calculate the attack rates by cluster. We estimated the attack rate within the sub-district by calculating the mean of attack rates for the 16 clusters combined. We also calculated the attack rates by 5-year age groups and gender. We used the number of suspect cases instead of confirmed cases to estimate the attack rate because we only collected and tested the serum samples from a sub-set of suspect cases who later came to clinics for clinical assessment and sample provision. For the sub-set of patients who came to the clinics for clinical evaluation, we described their clinical features. Since many respondents were still symptomatic during the clinical survey, we estimated the median follow-up time from symptom onset till recovery for each of the four symptoms including joint pain, joint swelling, debilitating weakness and severe myalgia. Previous authors have suggested that length of follow-up time should be extracted from the time frame where the Kaplan Meier estimate was most stable.[46] We considered the longest median of 31 days as the follow-up time before applying right censoring to generate Kaplan-Meier curves for describing the median durations of persistence of each of these symptoms that likely interfered with routine activities.[46]

### Ethical considerations

The Institutional Review Board (IRB) of IEDCR and the Ethical Review Committee (ERC) of icddr,b considers outbreak investigations that primarily aim to control outbreaks as public health practice and approval by research ethics committee is not required. This is in line with national and international ethical guidelines including the 1991 International Guidelines for Ethical Review of Epidemiological Studies by the Council for International Organizations of Medical Sciences.[47–49] Several published reports of outbreak investigations in the past have also reported similar exemption from ethical clearance.[50–52] While we agree that the boundary between public health research and practice remains poorly defined, this outbreak investigation was judged to be a routine public health activity (part of an emergency public health response), and therefore determined to not involve human subjects research and exempted from ethical review. However, all outbreak investigations follow the regulations on patient privacy and protection as stated in existing national and international guidelines including the Bangladesh Outbreak Manual.[49, 53] This investigation was approved by and conducted in collaboration with the Government of the People's Republic of Bangladesh.

We obtained informed oral consent for voluntary participation from adults and verbal assent from children. Past experience of research in similar settings suggested that obtaining written informed consent was difficult, time consuming and resource-intensive when dealing with illiterate participants who were often very cautious, as they did not know what they were signing or whether the signed document could be used against them. As a result, verbal informed consent procedure was allowed during this emergency outbreak situation, where the majority of the affected population were illiterate and ill, as obtaining written informed consent was considered unnecessary and not in the patient and community’s best interest. The investigation team member who was informing a participant signed a form stating that the appropriate information was provided and verbal consent received before beginning any information and/or sample collection or any kind of investigation.

## Results

### Epidemiological findings

#### Preliminary investigation

The team identified 30 suspect cases from the hospital initially. These cases resided in six different unions within Shibganj. Their median age was 35 (Inter-quartile range, IQR: 49−30) years and 45% were males. Fever (100%), polyarthralgia (90%), malaise (83%), severe weakness (66%), rash (48%), itching (38%), and headache (35%) were the predominant symptoms. Forty percent (12/30) of these patients had evidence of IgM CHIKV antibodies in their sera when tested using the rapid assay.

#### Findings from the syndromic survey

We surveyed a total of 5,902 individuals from 1,283 households in the 16 unions of Shibganj. Of these, 1,769 (30%) met the suspect case definition. The median age of suspect cases was 28 (IQR:15−42) years. The average attack rate in the sub-district was 30% (95% CI: 27%−33%). Attack rates varied from 10%−44% across the 16 selected clusters within the 16 unions of Shibganj (Table 1). The mean attack rate of 33% in females (95% CI: 27%−39%) was higher than in males (29%, 95% CI: 23%−34%). However, the mean attack rates did not vary significantly by gender (P = 0.326). Children <5 years of age (15%) were least likely to be affected when compared to the individuals in the other age-groups (Table 2). In addition to fever, 99% (1747/1769) of the suspect cases reported suffering from joint pain, and 29% (514/1,769) from rash.

**Table 1:** Attack rates of suspected chikungunya fever cases per cluster in the 16 unions of Shibganj sub-district, Chapainababganj District, Bangladesh, 1 September –15 December, 2011.

Union name	Attack Rate (%)	Confidence Interval, 95% CI
**Pourashava**	34.4	24% - 36%
**Binodpur**	27	
**Chakkirti**	44	
**Daipukuria**	10	
**Dhainagar**	16	
**Durlovpur**	29	
**Ghorapakhia**	33	
**Mobarakpur**	24	
**Monakasha**	38	
**Noyalavanga**	40	
**Panka**	13	
**Chhatrajitpur**	37	
**Shahabajpur**	41	
**Shyampur**	43	
**Kansat**	22	
**Ujirpur**	30	

**Table 2:** Attack rates of suspected chikungunya fever cases by age group and gender in Shibganj sub-district, Chapainababganj, Bangladesh, 1 September and 15 December, 2011.

Age groups	Attack rates		
	Proportion of suspect cases (%)	Proportion of male suspect cases (%)	Proportion of female suspect cases (%)
**0–4**	81/527 (15)	40/258 (15)	41/268 (15)
**5–9**	162/695(23)	79/354 (22)	83/341 (24)
**10–14**	219/765 (29)	115/371 (31)	104/394 (26)
**15–19**	194/634 (31)	102/321 (32)	92/313 (29)
**20–24**	153/560 (27)	71/260 (27)	82/300 (27)
**25–29**	167/539(31)	66/261 (25)	101/278 (36)
**30–34**	173/524 (33)	61/241 (25)	112/283 (40)
**35–39**	155/408 (38)	72/211 (34)	83/198 (42)
**40–44**	138/401(34)	80/228 (35)	58/173 (34)
**45–49**	117/311(38)	63/177 (36)	54/134 (40)
**50–54**	101/267 (39)	49/135 (36)	52/132 (39)
**55–59**	52/160 (33)	21/74 (28)	31/86 (36)
**60–64**	63/190 (33)	36/104 (35)	27/86 (31)
**65–69**	44/121 (36)	22/54 (41)	22/67 (33)
**70–74**	22/88 (25)	13/56 (23)	9/32 (28)
**75–79**	8/27 (30)	4/20 (20)	4/7 (57)
**80–84**	5/21 (24)	4/8 (50)	1/13 (8)
**85–89**	1/5 (20)	0/3 (0)	1/2 (50)

#### Serological findings

Among the 1,769 suspect cases, 480 (27%) were invited to participate in the clinical survey and to provide blood samples. Seventy-nine percent (377/480) of the invited suspect cases gave consent for the clinical survey and physical examination, and 367 (77%) provided a blood sample. Among the serum samples tested with the rapid test, 73% were positive for chikungunya whereas IgM anti-CHIKV antibodies were detected in 78% (264/338) of the samples that were tested by ELISA. We found that the suspect cases tested within one week of illness onset (43%) were less likely to have IgM antibodies against Chikungunya compared to the suspect cases tested more than 60 days post illness onset (98%) (S1 Table).

#### Findings from the clinical survey

Among the 377 cases interviewed during the clinical survey, the median age was 40 years (IQR:30−50 years); 64% (241/377) were females; and 92% (347/377) were Muslims. The main clinical symptoms included fever (100%), joint pain (88%), debilitating weakness preventing routine activities (55%), rash (51%), severe myalgia (44%), itching (33%) and joint swelling (16%) (Table 3). Thirty-four percent (128/377) of the cases reported presence of large numbers of mosquitoes in the areas where they had lived before becoming sick, and 27% (102/377) lived in households with at least one other suspect case. Among the suspect cases who had visited the clinic, 57% (215/377) were still symptomatic during the survey. Detailed information collected from the sub-set of patients during the clinical survey suggested that the outbreak may have lasted for more than three months and had peaked in early November (S1 Fig).

**Table 3:** Clinical features of suspect cases of chikungunya fever during the outbreak in Shibganj Sub-district of Chapainababganj District, Bangladesh in 2011.

Symptoms	Total suspected CHIKF cases (%) (n = 377)
**Fever**	377 (100)
**Joint pain**	331 (88)
**Weakness interfering daily activities**	206 (55)
**Rash**	192 (51)
**Debilitating myalgia**	165 (44)
**Itching**	123 (33)
**Malaise**	136 (36)
**Headache**	111 (29)
**Joint swelling**	59 (16)
**Vomiting**	28 (7)
**Cough**	27 (7)
**Sore throat**	26 (7)
**Abdominal pain**	21 (6)
**Difficult breathing**	13 (3)
**Convulsion**	6 (2)
**Bleeding from the skin/mucosa**	7 (2)

**Fig 2:**
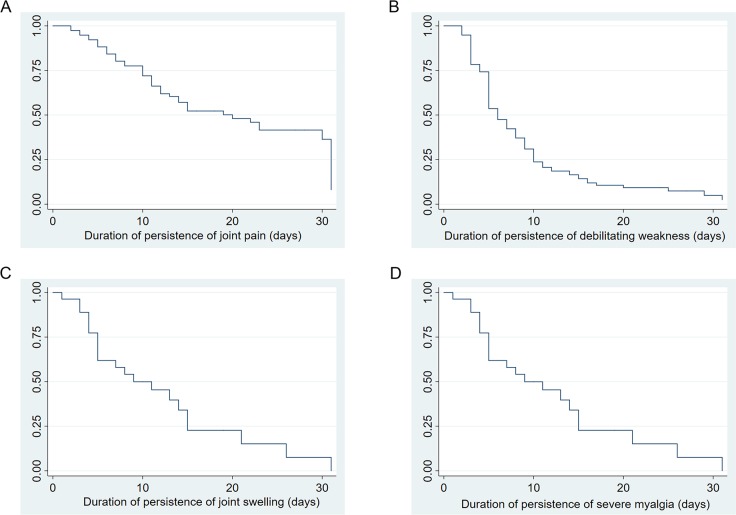
Kaplan-Meier survival curves showing the durations of symptom persistence among serologically confirmed chikungunya cases (n = 264) who experienced those symptoms during the outbreak in Shibganj Sub-district of Chapainababganj District, Bangladesh in 2011. Fig 2A showing persistence of joint pain (n = 156), 2B showing debilitating weakness (n = 105), 2C showing joint swelling (n = 37), and 2D showing severe myalgia (n = 94).

In addition to fever, the predominant symptoms of serologically-confirmed cases (n = 264) among those who had provided sera after coming to the clinic included joint pain (97%), weakness (54%), myalgia (47%), rash (42%), itching (37%) and malaise (31%). The rashes were found on the trunk (36%), lower limbs (36%), palms and soles (33%), upper limbs in (14%), and face (8%). Of those who developed joint pain, 93% (238/256) reported pain in multiple joints and joints of the lower limbs were involved in 90% (230/256) of the cases. Among the cases reporting joint pain, severe pain that prevented sleep was reported by 61% (156/256), moderate pain that caused pain on movement was reported by 23% (59/256) and minor pain that did not interfere with daily activities but caused discomfort was reported by 17% (43/256). Severe weakness was reported by 40% (105/264), debilitating myalgia by 36% (94/264) and severe joint swelling by 14% (37/264). On examination, team physicians found restricted joint mobility in 23% (59/256) of the serologically confirmed cases. The rashes were maculopapular in 62% (69/112), macular in 33% (37/112) and popular in 5% (6/112) of the suspect cases with rashes. The median durations of symptom persistence of joint pain was 31 days, while joint swelling persisted for 7 days, debilitating weakness and severe myalgia, each persisted for about 12 days in the majority of this sub-set of confirmed cases (Fig 2). Furthermore, 18%, 11%, 7% and 3% of cases still reported suffering from severe joint pain, debilitating joint swelling, severe myalgia and severe weakness respectively after three months post illness onset (Table 4). We did not detect any deaths or sub-acute cases in this outbreak. The majority (79%) of the confirmed cases (209/264) sought care from the primary healthcare clinics, with only 6% seeking out-patient services from the sub-district hospital.

**Table 4:** Duration of symptom persistence in seropositive cases during the outbreak in Shibganj Sub-district of Chapainababganj District, Bangladesh, 2011.

Symptom	ELISA positive cases with symptom (n)	Total number (%) of cases with relief of symptom at 7 days	Total number (%) of cases with relief of symptom at 15 days	Total number (%) of cases with relief of symptom at 21 days post onset	Total number (%) of cases with relief of symptom at 30 days	Total number (%) of cases with relief of symptom at 45 days	Total number (%) of cases with relief of symptom at 90 days
**Severe weakness**	105	56 (53%)	85 (81%)	90 (86%)	95 (91%)	102 (97%)	102 (97%)
**Severe Joint Pain**	156	18 (11%)	49 (31%)	56 (31%)	70 (45%)	114 (65%)	128 (82%)
**Joint swelling**	37	12 (32%)	23 (62%)	26 (70%)	27 (73%)	32 (87%)	33 (89%)
**Severe myalgia**	94	35 (37%)	54 (57%)	54 (57%)	60 (64%)	77 (82%)	87 (93%)

#### Molecular characterization and phylogenetic analysis

**Fig 3:**
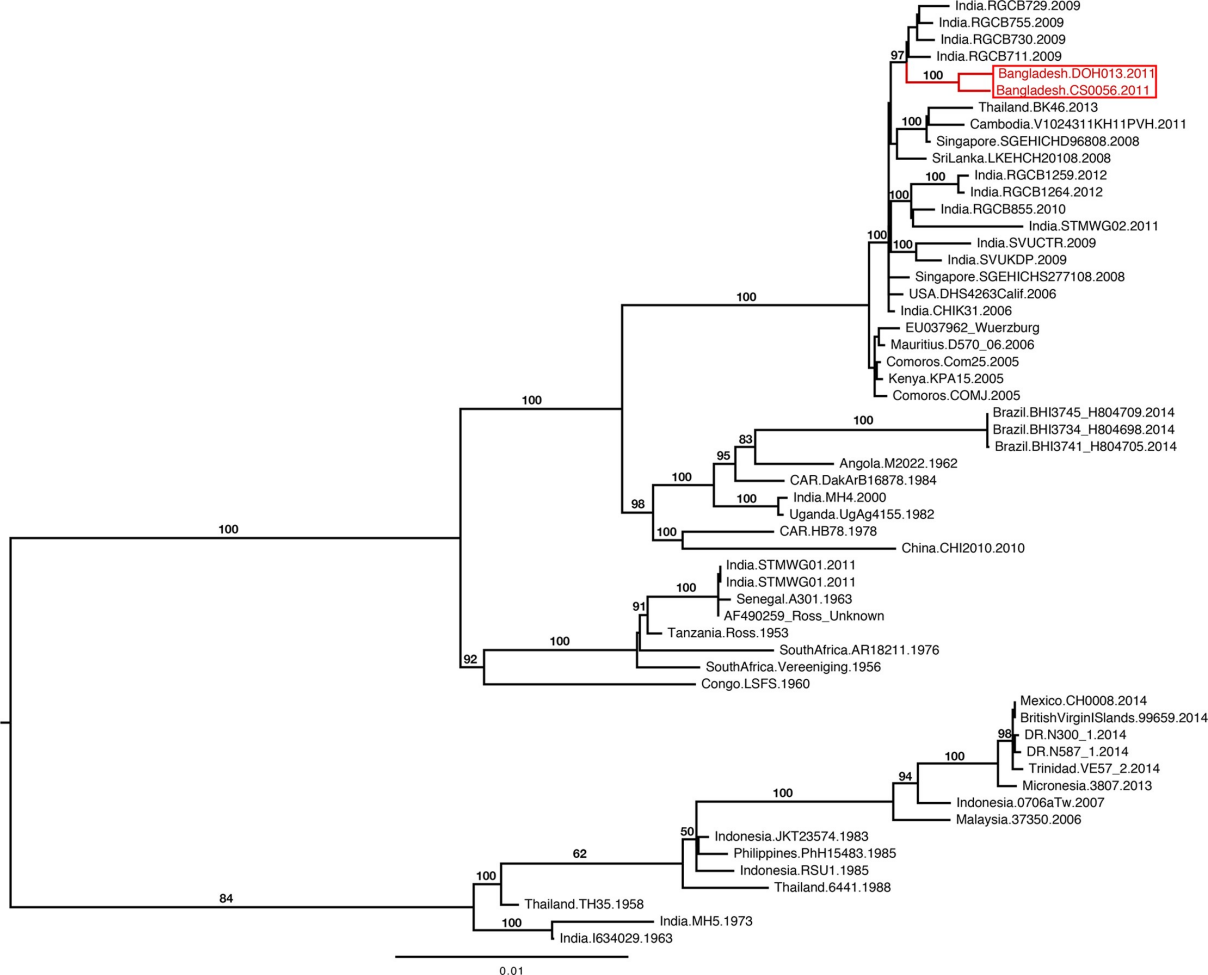
Maximum-likelihood phylogenetic analysis of the complete full-genome nucleotide sequence of CHIKV strains identified in Bangladesh during 2011 and in different parts of the world. Bootstrap support values >90% are indicated at the relevant nodes.

Two out of the four patients from this outbreak in Shibganj and the single patient from the concurrent outbreak in Dohar sub-district of Dhaka whose acute sera were collected within two days of illness onset had detectable Chikungunya virus RNA with RT-qPCR. However, none of them had IgM antibodies in their sera. The three viral isolates, obtained from the two outbreaks including this outbreak in Shibganj (n = 2) and in Dohar (n = 1), clustered with strains from the 2006 Indian Ocean lineage and were most closely related to the Indian/Asian geographic isolates rather than the African isolates within that lineage (Fig 3). The size of the genome was 11,825 bp in length. Molecular analysis identified mutations in E2 and E1 glycoproteins and contained the E1 A226V point mutation (S2 Table).

### Entomological findings

The team did not find any larvae of *Aedes aegypti* or *Aedes albopictus* mosquitoes during the investigation. The team found mosquito larva from only 1/160 households surveyed and the larvae that hatched from this household in IEDCR’s Entomology laboratory yielded *Culex quinquefaciatus* species.

## Discussion

We identified CHIKF suspect cases among 30% of the residents surveyed across the 16 unions in Shibganj during the syndromic survey. The median age of suspect cases was 28 years. The lowest attack rate was found in children <5 years (15%). Anti-chikungunya virus (CHIKV) IgM antibodies were detected by an enzyme-linked immunosorbent assay (ELISA) in 78% of the case samples tested. In addition to fever, the predominant symptoms of serologically-confirmed cases, included joint pain (97%), weakness (54%), myalgia (47%), rash (42%), itching (37%) and malaise (31%). The median durations of symptom persistence of severe joint pain was 31 days, while severe joint swelling persisted for 7 days, debilitating weakness and severe myalgia, each persisted for about 12 days in the majority of this sub-set of confirmed cases. Among the sero-positive patients, 79% sought healthcare from outpatient clinics. CHIKV was isolated from two cases and phylogenetic analyses of full genome sequences placed these viruses within the Indian Ocean Lineage (IOL). Molecular analysis identified mutations in E2 and E1 glycoproteins and contained the E1 A226V point mutation. We did not find any larvae of *Aedes aegypti* or *Aedes albopictus* mosquitoes during the investigation.

The clinical syndrome of fever, multiple joint pain, with or without debilitating weakness or severe myalgia was consistent with previous CHIKF outbreaks.[2, 3, 5, 33] The detection of IgM antibodies in the majority of the sera collected from suspect cases, and the isolation of CHIKV from suspect cases' sera, confirmed a febrile illness outbreak caused by CHIKV in Shibganj sub-district of the northwestern district of Chapainababganj in Bangladesh. This was the second recognized epidemic within the district that had caused clinical illness in one-third of the residents within a period of about three months,[31] and emphasized the multi-year risk of CHIKV epidemics in Bangladesh. Given that CHIKV infection confers life-long immunity,[14] the consistently high attack rates by age groups suggested that Shibganj’s residents had little previous immunity to CHIKV and, therefore, recent introduction of CHIKV in this community.

Though several outbreaks have reported an increased propensity to cause clinical illness among females,[54] our syndromic survey revealed no gender differences for acquiring CHIKV infection. However, similar to another outbreak, children <5 years of age seemed least likely to develop clinical illness compared to older individuals.[13] While, we did not find any deaths or cases with chronic comorbidities or cases that qualified as atypical, we did detect severe and chronic symptoms in this outbreak.[13, 25–30, 55] The prolonged persistence of debilitating symptoms including arthralgia and the consequent disruption of routine activities, constituted a considerable burden on the outbreak-affected community.

The first recognized outbreak in Chapainababganj district in 2008 predominantly affected Hindu potters and had limited geographic spread. The outbreak was reportedly precipitated by the high concentration of *Aedes albopictus* larvae breeding in the numerous wet earthen and clay pots that were used for pottery.[31] During the three years after the detection of the first chikungunya outbreak in Bangladesh, several minor CHIKV outbreaks, each of which affected a limited number of people but lasted for a duration similar to the current epidemic, were detected in the country. After resurgence in Kenya in 2004, explosive epidemics with attack rates of 40%−75%, and emergence in new geographic areas have been observed in the Indian Ocean islands, India, Malaysia, Indonesia and the Americas.[3, 14, 56] On the contrary, CHIKV transmission in Bangladesh have been limited in geographic scope and time span and ended without major prevention efforts,[31, 33] despite the apparently widespread presence of both types of mosquito vectors, high population density and frequent movement of people and goods to and from CHIKV-endemic neighbouring countries including India. We did not find any breeding places for the vector *Aedes* mosquito from the affected area, a finding quite inconsistent with past outbreaks. The delayed reporting of the outbreak with the consequent delay in conducting the investigation during the dry winter months of November and December possibly prevented us from detecting the vector. Given the role of environmental variables on *Aedes albopictus* and on the pathogen transmissibility, we believe that transmission likely stopped due to changes in weather that constrained *Aedes* mosquito populations during this outbreak.[57, 58] We were unable to find *Aedes* larvae by December, and other outbreaks in Bangladesh have also stopped abruptly around the months of November and December.[31, 33, 34] Virus isolates from this outbreak had a mutation in the position 226 of the gene for the membrane fusion glycoprotein E1,which has been shown to enhance viral capacity to infect and replicate in *Aedes albopictus* species.[59] Another outbreak identified in Bangladesh in 2011 identified that *Aedes albopictus* were most likely responsible for transmission,[33] and taken with evidence about the mutation we identified, suggests that this vector may have also been responsible for this outbreak.

A major limitation of our investigation was that the suspect cases that presented for clinical assessment and blood collection were unlikely representative of the entire affected population. We expect that cases with severe and/or persistent symptoms were more likely to come to the clinic compared to milder cases or patients who had recovered, so our clinical descriptions of serologically confirmed cases were likely biased towards severe presentations. However, since almost half of the cases had already recovered from clinical illness during the clinical survey, we also captured some less serious disease. Given that an evaluation of the sensitivity and specificity of clinical criteria for CHIKV infection identified the syndrome combining fever and polyarthralgias to allow for the correct classification of 87% of those who had serologically confirmed CHIKV infections,[60] our case selection criteria combining fever and joint pain or rash was considered reliable by the national expert team. A second limitation was that we used suspect case counts to calculate attack rates. This strategy may have led us to include cases of febrile illness that did not have CHIKV infection, leading to overestimation of cases. On the contrary, this strategy allowed the detection of symptomatic cases with acute disease only leading to possible underestimation of the number of people infected during the outbreak given evidence that 3–25% of chikungunya virus infections are asymptomatic.[36, 61] Thirdly, constrained laboratory resources limited our ability to collect and test samples from all suspected cases. Furthermore, the diagnostic tests that we used had less than optimum sensitivity and specificity. This suggest that we likely missed detection of some true cases. However, among suspect cases that were tested, a high proportion of them (264/338, 78%) did have IgM antibodies against CHIKV, and considering that one-fifth of the patients tested were more than two months post illness onset, it is likely that most of the suspect cases we identified did have CHIKV infections.

Though no recent community seroprevalence study of CHIKV has been published from Bangladesh, a post outbreak sero-survey conducted in selected outbreak affected communities under publication by the author group as well as a 1995 cross-sectional survey carried out in the neighbouring city of Calcutta, India indicated that the level of previous exposure to Chikungunya infection in the country remained low.[33] Furthermore, outbreaks were consistently reported in 2012 and 2017, including the epidemic spread detected in the Capital, Dhaka in 2017.[32] CHIKF outbreaks are likely to continue to occur in Bangladesh, where the underlying immunity likely remains low. However, surveillance efforts to detect these outbreaks would be difficult to implement given that many cases may never seek care, and those who seek care visit the primary care, out-patient clinics instead of the hospitals where chikungunya diagnostic capacity is limited. Indeed, given these limitations, many outbreaks have likely gone undetected in Bangladesh. Although the community impact of CHIKF outbreaks can be severe, because of lasting symptoms, evidence suggest that outbreaks here are less likely to be long-lasting because of seasonal reductions in *Aedes* populations.

## Conclusions

Population-based seroprevalence and risk factor surveys could help describe the true extent of previous circulation of this virus and identify factors that might put some communities at higher risk than others for outbreaks. Molecular clock analyses and monitoring the spread of genotype viruses and the proportion of the *Aedes* mosquitoes in the future may help provide important insight into emergence of epidemic strains in Bangladesh. Effective mosquito control using a comprehensive and integrated pest management approach targeted at every life stage of a mosquito could reduce the risk of CHIKV dissemination.[8, 13] To enhance global health security, implementation of better surveillance methods to rapidly identify communities at risk are needed to ensure adequate and effective outbreak response.

## Supporting information

S1 Table Proportion of suspected patients tested with evidence of IgM antibodies against Chikungunya virus in serum by days since illness onset. (DOCX)

S2 Table The mutations in the E1 and E2 proteins and containing point mutations at A226V. (DOCX)

S1 Fig Week of onset of illness among the suspect cases recruited for the clinical case survey. (DOCX)

S1 Text Poster and data availability information. (DOCX)

S1 Dataset Syndromic survey de-identified data in SPSS. (SAV)

S2 Dataset De-identified clinical survey data in SPSS. (SAV)

S3 Dataset De-identified symptom duration data in SPSS. (SAV)
