# Supplementary material for: An epidemic of chikungunya in northwestern Bangladesh in 2011
Source: PLoS One. 2019 Mar 11;14(3):e0212218. doi: 10.1371/journal.pone.0212218 (PMC6411100; doi:10.1371/journal.pone.0212218)
Supplement: S1 Table — (DOCX) [file pone.0212218.s001.docx]

**S1 Table. Proportion of suspected patients tested with evidence of IgM antibodies against Chikungunya virus in serum by days since illness onset, Shibganj, Chapainababganj, Bangladesh, September 1–December 15, 2011 (n=338).**

| **Days since illness onset** | **Number (Percentage positive)** |
| --- | --- |
| **<7** | 6 (43) |
| **7-15** | 16 (42) |
| **15-30** | 62 (77) |
| **30-60** | 65 (73) |
| **>60** | 115 (98) |
| **Total** | 264 (78) |
