# Supplementary material for: An epidemic of chikungunya in northwestern Bangladesh in 2011
Source: PLoS One. 2019 Mar 11;14(3):e0212218. doi: 10.1371/journal.pone.0212218 (PMC6411100; doi:10.1371/journal.pone.0212218)
Supplement: S2 Table — (DOCX) [file pone.0212218.s002.docx]

**S2 Table. The mutations in the E1 and E2 proteins and containing point mutations at A226V.**
