# Supplementary material for: An epidemic of chikungunya in northwestern Bangladesh in 2011
Source: PLoS One. 2019 Mar 11;14(3):e0212218. doi: 10.1371/journal.pone.0212218 (PMC6411100; doi:10.1371/journal.pone.0212218)
Supplement: S1 Fig — (DOCX) [file pone.0212218.s003.docx]

**S1 Fig. Week of onset of illness among the suspect cases recruited for the clinical case survey during the chikungunya fever outbreak in Shibganj sub-district of Chapainababganj District, Bangladesh in 2011 (N=377)**
