# Supplementary material for: An epidemic of chikungunya in northwestern Bangladesh in 2011
Source: PLoS One. 2019 Mar 11;14(3):e0212218. doi: 10.1371/journal.pone.0212218 (PMC6411100; doi:10.1371/journal.pone.0212218)
Supplement: S1 Text — (DOCX) [file pone.0212218.s004.docx]

**S1 Text: Poster and data availability information**

**S1A Text. Poster presentation at the 62nd ASTMH annual meeting**

Name of conference: Presented the abstract as a poster in the 62nd Annual Meeting of the American Society of Tropical Medicine and Hygiene (ASTMH), which was held during November 13-17, 2013 at the Marriot Wardman Park, Woodley Road, Washington DC, USA.

**S1B Text. Data availability information**

**S1 Dataset. Syndromic survey de-identified data in SPSS**

We have removed the personal identifiers and the GPS coordinates of the households to ensure anonymity and confidentiality.

**S2 Dataset. De-identified clinical survey data in SPSS**

We have removed the personal identifiers including the names of the participants and the household heads, dates of investigation, dates of onset of illness and the GPS coordinates of the households to ensure anonymity and confidentiality.

**S3 Dataset. De-identified symptom duration data in SPSS**

We have removed the personal identifiers and dates of start and end of symptoms also to further de-identify the data in accordance with the journal’s publication guidelines.
